# Flex multimode neural network for complete optical computation

**DOI:** 10.1016/j.isci.2025.112376

**Published:** 2025-04-08

**Authors:** Zeyu Deng, Zhangqi Dang, Ziyang Zhang

**Affiliations:** 1 Laboratory of Photonic Integration, School of Engineering, Westlake University, 18 Shilongshan Road, Hangzhou 310024, China

**Keywords:** Natural sciences, Physics, Applied sciences

## Abstract

Compact and efficient photonic integrated circuits (PICs) are promising route to solving modern computing challenges. Traditional PICs using cascaded Mach-Zehnder Interferometers (MZIs) or micro-ring resonators (MRRs) are limited to rigid linear matrix operations, requiring electronics for data compression, nonlinear activation, and post-processing. The dependence on electronic processing counteracts the advantages brought by photonics. Here we propose a photonic chip that tackles this problem. The idea is to apply two sets of electrodes on a multimode waveguide: one set for data loading and the other for shaping the neural network by manipulating the multimode light interference flexibly. The shaping process, following a genetic algorithm, resorts again to optical computation to bypass the gradient acquisition problem. Once trained, the chip handles computation completely in the optical domain. Experimentally 91% classification accuracy is achieved on the Iris dataset. Our approach may bring PICs closer to practical computation applications without electronics overload.

## Highlights

• A new, chip-based architecture for optical computation is established
• Multimode light interference is shaped by AI technique toward neural network computation
• The gradient acquisition problem in AI processing is solved by optical computation
• This simple photonic chip can handle 100% computation without any electronic processing

## Graphical abstract

**Figure:**
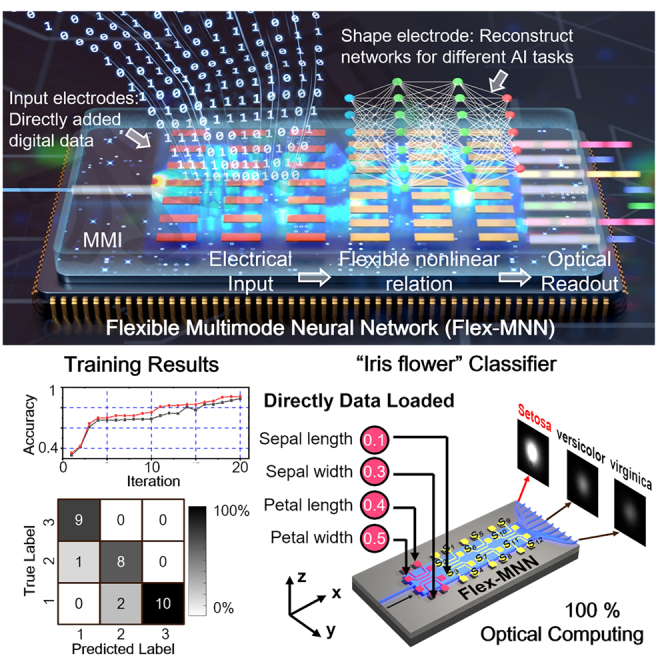

## Introduction

Optical computing has emerged as a promising alternative to perform neural network (NN) computations, owing to the inherent parallelism of light, low power consumption, and high-speed capabilities.^1^ This technology has drawn significant interest for deep learning applications, such as image classification^2^ and voice recognition.^3^ Within the optical computing domain, reconfigurable and programmable photonic integrated circuits (PICs) have become a popular tool thanks to their compact size, high efficiency, and versatile functional properties.^4^^,^^5^^,^^6^ These PICs are typically constructed by cascading basic photonic elements, such as Mach-Zehnder interferometers (MZIs)^7^^,^^8^ or micro-ring resonators (MRRs),^9^^,^^10^ into extensive computing networks. These networks can be configured to control light paths, thereby manipulating the transmission matrix.

However, MZI and MRR networks operate predominantly in the linear regime, limiting their ability to perform only linear matrix operations. In advanced neural networks, a nonlinear activation function (NAF) is key, as it mimics the function of synapses in the brain’s nervous system.^11^ In a photonic NN, the NAF is not straightforward to implement. One solution relies on the conversion between the optical and electrical domains, known as O-E-O conversion, which typically involves integrating additional photodiodes and modulators into the photonic network.^12^^,^^13^ These extra components increase system complexity and may introduce serious crosstalk issues. The second solution operates entirely within the optical domain such as electromagnetic-induced transparency,^14^ phase change materials,^15^ and second harmonic generation,^16^ etc. Unfortunately, achieving optical nonlinearity generally requires high optical power and therefore poses a challenge to the system power management.^17^ To make it worse, the nonlinear activation responses are often fixed for the chosen material and cannot adapt to different applications.

Another challenge in advanced photonic computing is the often indispensable integration of the electronic layer. As the size of the single-mode optical computing network is limited, a fully connected electrical layer is often needed before the photonic chips to compress the data into an acceptable input vector^7^^,^^18^ (e.g., to convert an image containing 28 × 28 pixels to a 1 × 8 vector). The training algorithm is applied not only for configuring the tuning units on the photonic chip but also for adjusting the weights in the electronic layer. However, the dependence on electronic computing places the very need for photonic layers in question. In fact, in many reported technologies, electronic computing is indispensable and even dominates the task, while the photonic parts are only there not hindering the process but not convincingly useful either. Therefore, it is of great interest to explore the possibility of whether an optical chip can handle 100% of the computation task while leaving the electronics only for data loading and reading out.

In our previous work, we have found that in a thermally tuned multimode waveguide, the relation between the electronic input and the optical output can be described by a complex nonlinear deep neural network (DNN). We named this equivalent network as the multimode neural network (MNN).^19^ Based on this finding, the MNN can be first employed as a NN accelerator to represent the nonlinear layer within a larger network. A fully connected electronic layer (FCEL) that performs only linear matrix operations is then applied in front of the MNN to convert the digital data to a set of currents and hence the electrical-optical MNN (EO-MNN) is established. The EO-MNN can classify nonlinear datasets by direct optical readout. However, the EO-MNN structure still needs the assistance of the electronic layer for training and computing. In essence, the MNN, though complex and nonlinear, is a fixed network that cannot be adjusted as the size of the multimode waveguide and the locations of the electrodes are determined. As a result, the weight-adjusting task is left on the electronics side.

**Figure 1:**
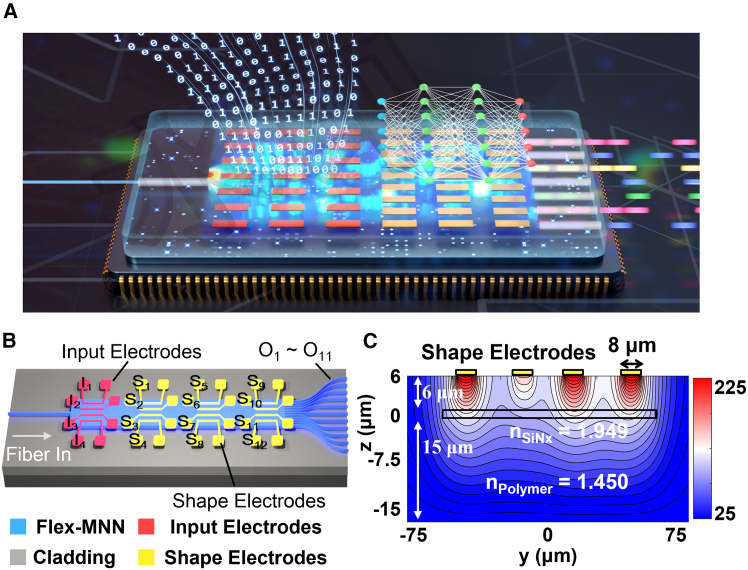
Architecture of the Flex multimode neural network (Flex-MNN) (A) Artistic illustration of the Flex-MNN. (B) Structure of the “Iris dataset” classifier. (C) Temperature gradient in the cross-section created by tuning on four electrodes placed on the top cladding.

To advance the technology, we propose a fully flexible multimode neural network and with this flexibility, we aim to bring optical computation to 100%. We name it Flex-MNN. The concept is illustrated in Figure 1A. This architecture involves a multimode waveguide and an electrode array that modulates the complex optical interference patterns within the waveguide. The key is that these electrodes are separated into two groups: one for data loading, i.e., they serve as the input electrodes and the other group works for network shaping, i.e., the shaping electrodes. Input electrodes are utilized to directly convey digital data from the given dataset without any processing, while the shaping electrodes are employed to dynamically adjust the multimode interference and in turn the structure of the MNN, thereby defining the number of NN layers and the numbers of neurons in each layer to best solve a given task.

It must be noted that the configuration in Figure 1B works favorably on thermally insulating optical materials such as polymer and glass, where the heaters can effectively generate a temperature gradient inside the material, as shown in Figure 1C. This temperature gradient is translated to local refractive index changes via thermo-optic effect. In thermally conducting materials, such as silicon, the waveguide tends to reach a uniform temperature, and it is inefficient to generate a strong gradient that affects the modes significantly. Previously, this index manipulation technique has led to the development of large-scale optical switches by thermo-optic waveguide lens.^20^ The interference shaping effect was also implemented for the invention of a nonlinear function generator,^21^ where the E-O response from one electrode to one waveguide output is defined by the other three electrodes to follow a target mathematical function. These technologies have laid the groundwork for this investigation.

In Figure 1B, once the Flex-MNN is trained, the computation results can be directly readout at the waveguide outputs. As proof of concept, we propose the “Iris dataset” classifier using the multimode device with 11 output ports and 16 electrodes, as shown in Figure 1B. Since the Iris flower comprises only three distinct categories, we have first selected 3 output ports for classification. The electrodes are then allocated as 4 input electrodes and 12 shape electrodes, respectively. The electrodes are divided into 3 groups, which represent 3 layers of the network, and each group contains 4 electrodes. More layers would raise the difficulties in training the Flex-MNN. The multimode waveguide is determined to have a width of 110 μm, which supports more than 100 modes in total. The number of supported modes should be larger than the equivalent neurons inside the Flex-MNN so that they can be efficiently adjusted for the tasks. The Flex-MNN is then trained on our homemade function programmable waveguide engine^22^ (FPWE) and the shape electrodes are adjusted through a genetic algorithm (GA). This is because the mathematical gradient is difficult to obtain inside the waveguide without the capability to monitor the internal light field distribution experimentally. It is noteworthy that although the GA optimization operates in the electronic domain during the training process, the forward computation is taken optically, and in the end, the optical computation within the Flex-MNN still accounts for 53.1% of the total calculations compared to a fully electronic NN.

After the shape electrodes are trained, the data can be loaded onto the input electrodes in the form of currents and light only needs to propagate through the waveguide once to obtain the computation results. The computation is completely performed in the optical domain. Further conversion of the results in the electronic domain, e.g., processing of the photocurrents, involves a simple comparison process requiring just two floating-point operations in the computer. The Flex-MNN device can dynamically adjust the structure of the NN to enhance the training accuracy but also works without the need for pre-processing from an electronic layer. We believe this approach demonstrates the potential of the Flex-MNN as an optical computing unit in solving AI problems. It may also trigger the invention of advanced photonic devices for communication, sensing, and astronomy.

## Results

### Design and fabrication of the Flex-MNN chip

Following the design in Figure 1C, the Flex-MNN chip is fabricated using a silicon nitride (SiNx) strip embedded in polymer, as this waveguide features a relatively compact size, requires only simple fabrication steps, and offers simultaneously high thermo-optic coefficient and low thermal conductivity.^23^ The fabrication follows the same process as described in the previous work.^24^

**Figure 2:**
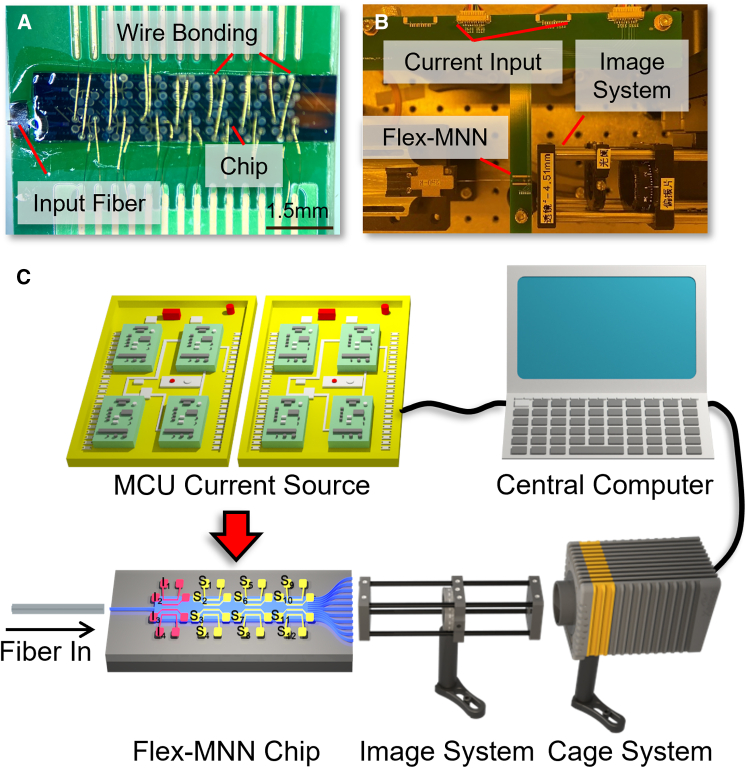
Photos of the chip integration and diagram of the function programmable waveguide engine (FPWE) (A) Microscope photo of the wire-bonded Flex-MNN chip on PCB. (B) Photo of the core O-E part in the FPWE system. (C) Diagram of the experimental setup.

After fabrication, the chip is wire-bonded to a Printed Circuit Board (PCB) and the input fiber is glue-fixed with the input single-mode waveguide. The assembly is shown in Figure 2A. Once assembled, the Flex-MNN chip undergoes training on the FPWE system.^22^ Detailed views of the optoelectronic assembly and the entire system are presented in Figures 2B and 2C, respectively. The thermo-optic response of the polymer material occurs on the millisecond level, allowing fast experiments and collection of large amounts of data.

### Training Flex-MNN using genetic algorithm

In a traditional NN, the weights are updated through backward propagation. This process calculates the gradient of the loss function for each neuron and then the derivatives propagate backward using the chain rule to pass the gradient layer by layer. However, the backward propagation is not straightforward to realize in optics. Though in principle it is possible to monitor the light field distribution inside the multimode waveguide, e.g., by scanning near-field microscopy, such a system becomes cumbersome to implement in practice. Instead, we choose the non-gradient-based GA to train the shape electrodes without the need for internal field tracking. This method, though not as efficient in the traditional sense as the gradient-based algorithm, can benefit from the fact that the computation in the training stage itself is still majorly performed in the optical domain, thus dramatically reducing the workload on the electronic side.

**Figure 3:**
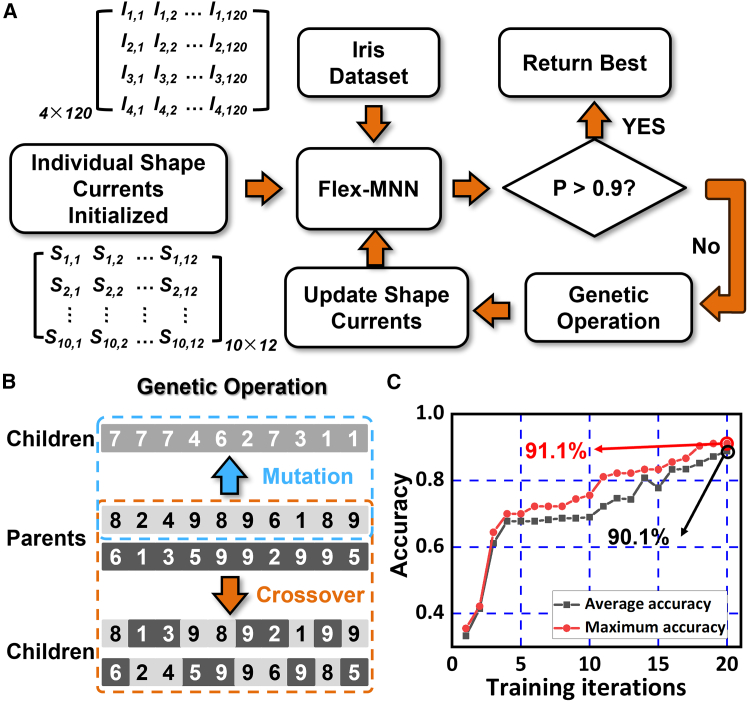
Working flow of the genetic algorithm (GA) and the training result (A) Diagram of the GA. (B) Diagram of the mutation and the crossover process in the genetic operation. (C) Accuracy evolution during the training process.

The GA is inspired by the principle of evolutionary theory, where individuals with higher adaptability to environmental variables are more likely to survive and reproduce.^25^ In our experiment, total 10 individuals are set and two individuals with the lowest classification accuracy undergo mutation, while the two with the highest accuracy are carried over to the next iteration unchanged. The remaining six individuals are subjected to the crossover operation. Besides, the threshold P (average accuracy of the 10 individuals) of the program is determined as 0.9. When P goes below this value, the GA continues to the genetic operation phase, as shown in Figure 3B. The training result is shown in Figure 3C, indicating that the P value reaches 90.1% after 20 iterations and the highest training accuracy goes up to 91.1% after convergence.

### Iris dataset classifier implemented by the trained Flex-MNN chip

**Figure 4:**
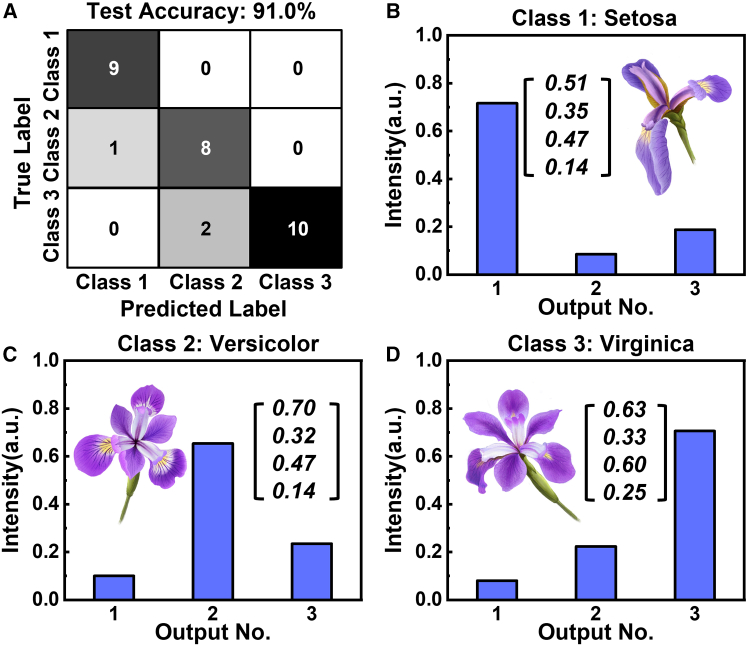
On-chip testing results (A) Confusion matrix of the experiment results on the FPWE system. (B–D) The computation results with measured intensity distribution for “Setosa,” “Versicolor,” and “Virginica,” respectively.

The testing data are used to validate the training results of the Flex-MNN, with the accuracy shown in Figure 4A. In our experiment, a total of 90 Iris samples are used for training, while the remaining 30 were used for testing. The test accuracy reaches 91% after 20 training iterations on our FPWE system. Figures 4B–4D illustrate the relative output spots for different types of Iris flower input, where the light power is predominantly concentrated in the target output port, thereby enhancing the classification accuracy of the testing data.

**Figure 5:**
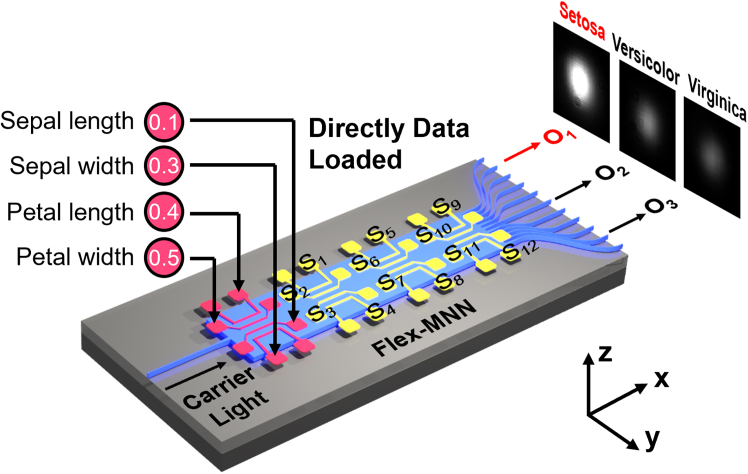
Working principle of the “Iris” classifier chip

Once the shape electrodes are configured, the data can be loaded onto the input electrodes in the form of currents and light only needs to propagate through the waveguide once to generate the computation results. Figure 5 illustrates the operation process of the “Iris dataset” classifier. Key values that describe the Iris flowers are first extracted, i.e., the sepal length, the sepal width, the pedal length, and the pedal width. These four values are converted to currents and applied to the input electrodes via the microcontroller unit (MCU). The data loading process is then complete. The currents on the shaping electrodes are set in a way that light propagates within the multimode waveguide under a special pattern to fulfill the computation task. The “shaping” of the Flex-MNN chip is also complete. Finally, light only needs to propagate over this chip to give the classification results, i.e., the output waveguide port with the brightest light spot concludes with the flower type. For the optional conversion of the results in the electronic domain, the computer only needs to read the intensity of the output light spots and determine the brightest one through two floating-point operations (comparison).

The actual operations of the optical computation, during training as well as in the testing/application stage, equivalent to the effective electronic computations, are summarized in Table S1. The traditional electronic NN that can handle the task consists of 4 neurons in the input layer and 3 neurons in the output layer. This electronic NN adopts the “tan-sigmoid” function as the activation function and converges after 20 iterations. The training stage is divided into forward calculation and backward training processes. In particular, to finish the forward calculation, the electronic NN needs to perform 158,400 float-point operations (FLOPs). On the Flex-MNN, this forward calculation is completed entirely in the optical domain. For the gradient-based algorithm done in the electronic NN, the total number of the FLOPs goes up to 468,240. For the Flex-MNN, the electronic computing needs to cover the backward updating and therefore 139,800 FLOPs are required. The total number of FLOPs amounts to 298,200, including the equivalent computation in the optical domain. Based on these calculations, optical computing during the training process accounts for 53.1% of the total calculation.

In the testing/application stage, the electronic NN requires 66 FLOPs to finish the matrix manipulation, followed by additional 2 FLOPs for comparison operations to determine the flower type. In contrast, the Flex-MNN allows digital data to be directly applied to the electrodes. The computation process is carried out by light propagation and the result/the flower type is given by the brightest output port. No electronic computing operations are needed at this stage and the ratio of optical computing goes to 100%.

## Discussion

As a new type of optical neural network (ONN), the Flex-MNN is so far the only implementation capable of handling 100% optical computation to the best of our knowledge. The comparison to the literature is provided in Table 1. For the MZI-based solutions, the network is limited by the small number of input/output ports. As a result, optical computation accounts for only a small ratio (below 6%) of the overall task. The directional couplers (DCs) or MRR mesh architecture is applied to achieve linear convolution operations in a convolution neural network (CNN) and thus could reach up to a ratio of 30.3% and 11.3%, respectively. However, the ring resonator requires critical environmental control to stabilize the spectrum for a consistent central wavelength over time. Furthermore, the DC needs precise structural design and control of the fabrication process, especially in the coupled waveguide region, to reach the 3-dB splitting ratio. The diffraction optical neural network (DONN) utilizes slots or meta-structured arrays to greatly increase the complexity of the optical layer. This approach increases the ratio of optical computation from 10.1% up to 58.8%. However, DONN is still linear in nature and this approach still relies on electronics for nonlinear computation.

**Table 1:** Comparison of the optical computing ratios for different ONNs

Reference	Year	Architecture	PIC complexity	Total number of FLOPs	Equivalent number of optical FLOPs	Ratio of optical computing
Zhang et al.^8^	2021	MZI mesh	28 MZIs + 56 electrodes	25,536	128	0.5%
Zhu et al.^18^	2022	MZI mesh + diffraction cell	20 MZIs + 2 diffraction cells + 40 electrodes	14,568	800	5.5%
Feldmann et al.^26^	2021	(De)Mux + DC mesh	4 MZIs + 16 MRRs + 16 PCMs + 40 electrodes	67,278	20,412	30.3%
Xu et al.^27^	2022	MZI Mux + MRR mesh	8 MZIs + 12 MRRs + 28 electrodes	1.64 × 10^7^	1.86 × 10^6^	11.3%
Fu et al.^28^	2023	Diffraction unit array	210 diffraction units + 10 electrodes (data added only)	38,280	22,400	58.5%
Cheng et al.^6^	2024	Trainable diffraction unit array	16 MZIs + 80 diffraction units + 96 electrodes	27,776	2,688	9.7%
This work	2025	Flex-MNN	1 multimode waveguide + 16 electrodes	68	68	100%

In contrast, our proposed Flex-MNN directly loads data onto the electrodes, completes both linear and nonlinear operations within the optical domain and obtains the computation results through optical readout. Furthermore, the design of the multimode optical chip is remarkably simple, consisting of just one multimode waveguide with 16 electrodes. To address more complex tasks, the waveguide size and the number of electrodes can be expanded to incorporate more input/shape electrodes as well as more output ports for readout. It must be stressed that in Flex-MNN the number of the input electrodes scales linearly with the number of the input data (N). In contrast, for the single-mode-based network, e.g., MZIs and MRRs, the number of electrodes scales with N^2^. Besides, the Flex-MNN can expand the multimode region allowing light to interfere diversely within a common region, while the single-mode-based approach would require a large routing mesh with many crossings that deteriorate the light signals gradually. Therefore, we believe the Flex-MNN technology may prove more efficient and robust in handling. The number of control units can also be flexibly increased to handle larger-scale neural computational applications.

To conclude, we have presented the concept of Flex-MNN and demonstrated that a small waveguide chip can indeed reach a 100% optical computation ratio, free from electronic computing in the application stage. The Flex-MNN is enabled by a multimode waveguide and a series of electrodes. The key is to divide the electrode array into two groups: the “normal” input electrodes that handle the direct loading of digital data onto the chip and the “tuning” shape electrodes that dynamically adjust the multimode interference toward an optimized, equivalent NN that tackles the task. As proof of concept, the standard “Iris dataset”^29^ is taken and the Flex-MNN fulfills the classifier function with 3 output ports and 16 electrodes (4 input and 12 shape electrodes). The GA is employed to train the Flex-MNN chip in order to avoid the complexity of obtaining the gradient information in the optical domain. During the training process, optical computation within the Flex-MNN accounts for 53.1% of the total calculations. In the application stage, this value goes to 100%. We therefore believe this powerful index tuning technology may bring the PIC-based ONNs closer to practical applications and may inspire the invention of advanced photonic devices in other fields such as communication, sensing, and astro-photonics.

### Limitations of the study

The Flex-MNN demonstrated in this work operates by the thermo-optic effect, which responds on the millisecond to microsecond level.^30^^,^^31^ Nevertheless, other index-tuning methods such as the ultrafast electro-optic effect, which operates on the nanosecond level,^32^ can also be adopted to modulate the local index in a multimode waveguide and induce the change of the multimode interference pattern. Moreover, as the input data volume and task complexity increase, the size of the multimode waveguide must be expanded, along with more input and shaping electrodes. The advantage is that the number of the input electrodes scales linearly with the number of the input data (N) but the single-mode-based network, e.g., MZIs and MRRs, scales with N^2^. Further studies are needed, however, to draw a clear relation between the waveguide dimension, in terms of supported mode numbers, and the dimension of the AI problem itself, in order to design the multimode waveguide more efficiently.

## Resource availability

### Lead contact

Further information and any requests should be directed to and will be fulfilled by the lead contact, Prof. Ziyang Zhang (zhangziyang@westlake.edu.cn).

### Materials availability

This study did not generate new unique materials.

### Data and code availability

Iris dataset data used in this article have been deposited at Mendeley Data (http://dx.doi.org/10.17632/fh3cn7pc4f.1) and are publicly available as of the date of publication. Accession numbers are listed in the key resources table. All data and code reported in this article will be shared by the lead contact upon reasonable request.

## Acknowledgments

This research received no external fundings.

## Author contributions

Conceptualization, Z.Z., Z. Dang, and Z. Deng; methodology, Z. Dang and Z. Deng; experiment, Z. Deng and Z. Dang; writing – original draft, Z.Z. and Z. Deng.

## Declaration of interests

The authors declare no competing interests.

## STAR★Methods

### Key resources table

**Table:** 

REAGENT or RESOURCE	SOURCE	IDENTIFIER
**Deposited data**		

Iris flower dataset	This paper, Mendeley data	https://dx.doi.org/10.17632/fh3cn7pc4f.1

**Software and algorithms**		

Matlab R2022a	Mathworks	https://www.mathworks.com
Lumerical HEAT	Ansys	https://innovationspace.ansys.com/product/lumerical-heat-intro/
origin	Originlab	https://www.originlab.com/
3ds Max R2024	Autodesk	https://www.autodesk.com/products/3ds-max/overview

### Method details

#### Design and fabrication of the Flex-MNN chip

The Flex-MNN chip is fabricated using a silicon nitride (SiNx) strip embedded in polymer. The polymer cladding (ZPU-450, from ChemOptics Inc.) and the SiNx core have refractive indices of 1.45 and 1.949 at 1550 nm wavelength, respectively. The SiNx layer has a width of 110 μm and a thickness of 150 nm. The thickness of the top and bottom cladding is 6 μm and 15 μm, respectively. Both the input and the shape electrodes are designed with a length of 150 μm, a width of 8 μm, and a thickness of 100 nm. In the fabrication process, only standard technology such as contact lithography and plasma-enhanced chemical vapor deposition is used.

#### FPWE system setup

In the FPWE system, the continuous-wave laser at 1550 nm is adopted as the carrier light. A polarizer is inserted before the light is injected into the chip to select only the TM light for analysis. Without high-speed PDs at hand, we have built an imaging system to capture the light from the chip facet by an IR camera (Bobcat-640, 512 × 640 pixels, 16-bit resolution). After that, a custom-made microcontroller unit (MCU) from the ARM-based 32-bit STM32F730XX series supplies the currents and drives the thermal electrodes. The MCU is capable of providing a maximum of 16 current channels. The current (DC) can be varied from 0 to 20 mA with a minimum step of 1μA. The accuracy of the MCU-based current source stays at the level of ±0.1%. The FPWE system is synchronously controlled by the MATLAB script on the computer.

#### Operation principle of the genetic algorithm

In our experiments, each “individual” represents a set of 12 current values applied on the shape electrodes and the population consists of 10 such individuals. More individuals would greatly increase the training time while less could prevent the algorithm from converging. We denote the current values as $I_{m,n}\;\left(m\leq 10,n\leq 12\right)$, where m represents the number of individuals and the n is the number of shape electrodes within the $m^{th}$ individual. For the $m^{th}$ individual, 120 data (90 for training and 30 for testing) in the “Iris dataset” are loaded on the input electrodes and the corresponding classification accuracy $\alpha_{m}$ is obtained. During the training process, one iteration sequentially loads 10 individuals onto the shape electrodes and hence the average training accuracy P is obtained by $P=\frac{1}{10}\sum \alpha_{m}$. This average accuracy P, ranging from 0 (complete failure) to 100% (perfect classification), is one key performance parameter during the training process.

The updating flow of the GA is shown in Figure 3A. The 10 individuals of the shape currents are initialized and one of the individuals is first loaded on the Flex-MNN. Then the “Iris” dataset with the size of 4 × 120 (i.e., 4 values in each and total 120 data in the dataset) is sequentially loaded onto the input electrodes and hence the corresponding accuracy for each individual is drawn. Once the accuracy for all 10 individuals is computed, the average accuracy P is determined. The threshold is set as 90%, i.e., when P goes above this value, the GA program concludes, and the shape currents are set. When P goes below this value, the GA continues to the genetic operation phase, as shown in Figure 3B. The genetic operations include 2 processes: mutation and crossover. In the mutation process, all current values in one individual are completely changed, while the crossover operation involves randomly exchanging current values between 2 individuals. In our program, the two individuals with the lowest classification accuracy undergo mutation, while the two with the highest accuracy are carried over to the next iteration unchanged. The remaining six individuals are subjected to the crossover operation. The shape currents are then updated for the next iteration.

### Quantification and statistical analysis

The offline and online training of the Iris flower dataset is completed using MATLAB R2022b. The temperature gradient in the cross-section of the multimode waveguide is simulated by the LUMERICAL Heat analysis. Figures in the main text and supplementary PDF were produced by MATLAB R2022b, origin and 3ds Max.
