# Supplementary material for: Flex multimode neural network for complete optical computation
Source: iScience. 2025 Apr 8;28(5):112376. doi: 10.1016/j.isci.2025.112376 (PMC12032928; doi:10.1016/j.isci.2025.112376)
Supplement: Document S1. Table S1 [file mmc1.pdf]

iScience, Volume *28*

Supplemental information

Flex multimode neural network for complete optical computation

Zeyu Deng, Zhangqi Dang, and Ziyang Zhang

Supplementary Tables

Table. S1. Breakdown of the computing process for the Iris Dataset.

<table>
<tr><th>Architecture</th><th>Processing stage</th><th>Calculation process</th><th>Implementation</th><th>Number of FLOPs</th><th>Total number of FLOPS</th><th>Ratio of optical computing</th></tr>
<tr><td rowspan="3">Equivalent electronic NN
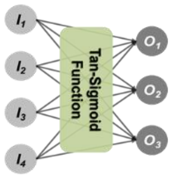

4 Input → 3 Output</td><td rowspan="2">Training</td><td>Network forward calculation</td><td>Electronic</td><td>158,400</td><td rowspan="2">468,240</td><td rowspan="3"></td></tr>
<tr><td>Weight backward updating</td><td>Electronic: Gradient based algorithm</td><td>309,600</td></tr>
<tr><td>Testing / Application</td><td>Network forward calculation</td><td>Electronic</td><td>68</td><td>68</td></tr>
<tr><td rowspan="3">Flex-MNN
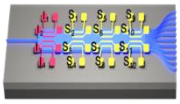
4 Input electrodes
12 Shape electrodes
3 Output</td><td rowspan="2">Training</td><td>Network forward calculation</td><td>Optical</td><td>Equivalent to 158,400</td><td rowspan="2">Equivalent to 298,200</td><td rowspan="2">53.1%</td></tr>
<tr><td>Shape electrodes backward updating</td><td>Electronic: Genetic algorithm</td><td>139,800</td></tr>
<tr><td>Testing / Application</td><td>Network forward calculation</td><td>Optical</td><td>Equivalent to 68</td><td>Equivalent to 68</td><td>100%</td></tr>
</table>

FLOPs: Float-point operations; *NN*: Neural Network
